# Supplementary material for: NET4 Modulates the Compactness of Vacuoles in Arabidopsis thaliana
Source: Int J Mol Sci. 2019 Sep 25;20(19):4752. doi: 10.3390/ijms20194752 (PMC6801981; doi:10.3390/ijms20194752)
Supplement: Supplementary file 1 [file ijms-20-04752-s001.zip › ijms-589832-SI-to conversion/ijms-589832-SI-to conversion.docx]

**Supplementary materials:**


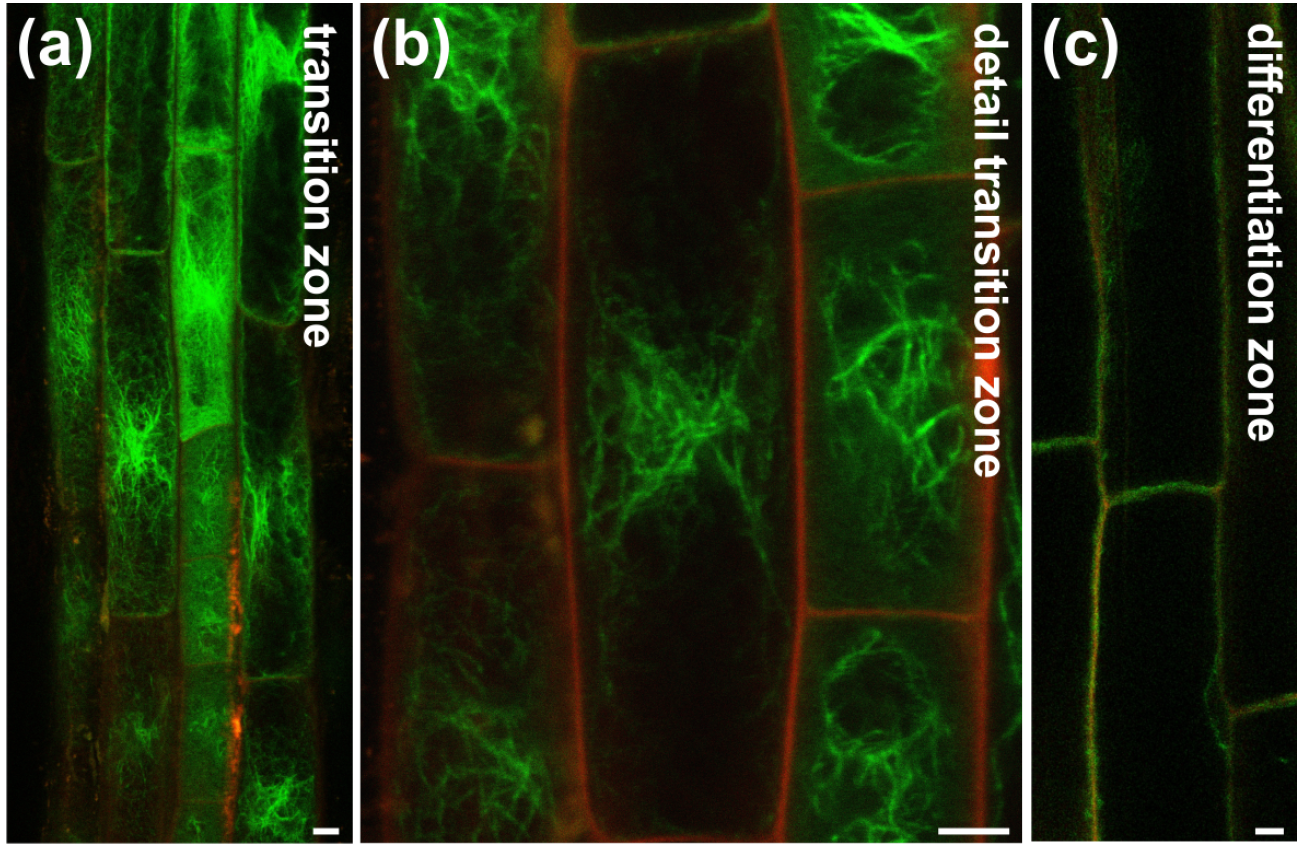


**Figure S1.** Spatial localization of NET4A. NET4A-GFP expression under its endogenous promoter in Arabidopsis roots. Expression starts in the late meristem, initially only in trichoblast cells (Figure 1a). (**a** and **b**) In (larger) atrichoblast cells, expression starts in the transition zone and continuous until the elongation zone. (**c**) NET4A-GFP is only weakly expressed in the differentiation zone. Propidium iodide (red) was used to stain cell walls. Scale bars: 5 μm.


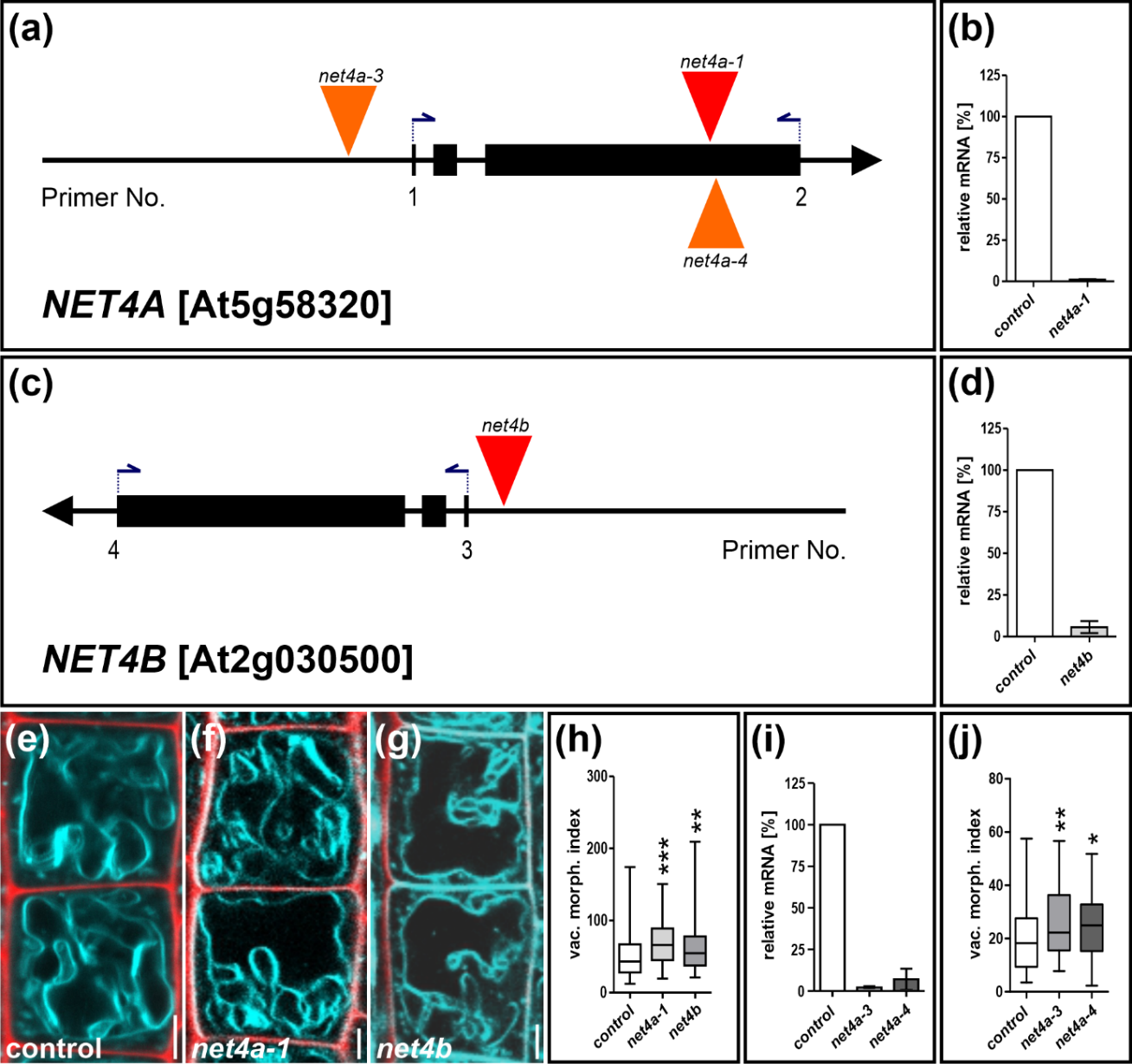


**Figure S2.** Loss-of-function mutants of *NET4A* and *NET4B*. **(a**-**d)** T-DNA insertion sites of *net4a-1* (SALK_017623), *net4a-3* (SALK_010530C), *net4a-4* (SALK_083604C) **(a)** and *net4b* (SALK_056957) **(c)** together with the according qRT-PCR to test for gene expression levels of *net4a-1* **(b)** and *net4b* **(d)**. Red arrowheads show verified, orange arrowheads predicted sites of insertion. Arrows indicate position of primers. Primers used are listed in supplemental table S1. **(e**-**h)** Vacuolar morphology of Col-0 control (*n* = 126), the *net4a-1* (*n* = 54) and the *net4b* single mutant (*n* = 99). MDY-64 (cyan) was used to stain vacuoles, propidium iodide (red) to stain cell walls. **(i)** qRT-PCR to test for gene expression levels of *net4a-3* and *net4a-4*. **(j)** Quantification of vacuolar morphology for Col-0 control (*n* = 64), the *net4a-3* (*n* = 63) and the *net4a-4* single mutant (*n* = 67). Columns of bar charts represent mean values, error bars represent s.e.m. Box limits of boxblots represent 25th percentile and 75th percentile, horizontal line represents median. Whiskers display minimum to maximum values. Student’s t-test, *p*-values: * *p* < 0.05; ** *p* < 0.01; *** *p* < 0.001. Scale bars: 5 µm.


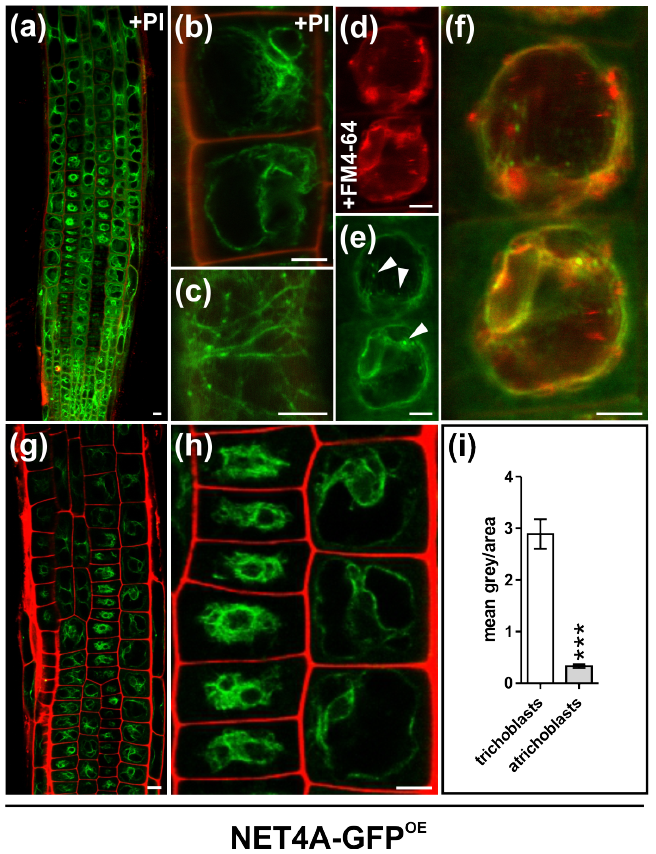


**Figure S3**. Localization of NET4A driven by the CMV 35S promotor (NET4A-GFPOE). (**a**) Uniform signal distribution within the Arabidopsis root meristem. (**b**) Vacuolar signal in atrichoblast cells and (**c**) filamentous signal at the cell cortex. (**d**-**f**) Vacuole staining by FM4-64 (3 h) shows NET4A colocalization at the tonoplast. White arrowheads highlight punctate signals. (**g**-**i**) More constricted vacuoles in trichoblast cells (*n* = 34) show a higher signal accumulation per area in comparison to less folded vacuoles in atrichoblast cells (*n* = 35). Propidium iodide was used to stain cell walls, FM4-64 to stain the tonoplast. Error bars represent s.e.m. Student’s t-test, *p*-values: *** *p* < 0.001. Scale bars: 5 μm.

Table S1: Used primers.

| **Gateway Cloning Primers** | **Sequence** |
| --- | --- |
| NET4A_FW | GGGGACAAGTTTGTACAAAAAAGCAGGCTTTATGGATTATGATCTGCTTCGTTC |
| NET4A_REV | GGGGACAAGTTTGTACAAAAAAGCAGGCTTAAGAAGCAAGAATGGATGATG |
| **Genotyping Primers** | **Sequence** |
| SALK_LBb1.3 | ATTTTGCCGATTTCGGAAC |
| SALK_017623(S1)_FW | AATGGATGATGGTCTTGTTGG |
| SALK_017623(S1)_REV | GAACACTGAGAGCTTGTTGCC |
| SALK_056957_net4b_FW | AATCACGATAGAGCCACATGC |
| SALK_056957_net4b_REV | TACATGCGGTAGAATTCCTCG |
| SALK_010530C(S3)_FW | TAGTCGCTTACACATTGGCAG |
| SALK_010530C(S3)_REV | TGAGAATTCGGTTGTTTTTGG |
| SALK_083604C(S4)_FW | TCCGGGTTGTTAGCTGTACAG |
| SALK_083604C(S4)_REV | CATCTGGGTTGGATGATGAAC |
| **qRT-PCR Primers** | **Sequence** |
| UBQ-5_fw | GACGCTTCATCTCGTCC |
| UBQ-5_rev | GTAAACGTAGGTGAGTCCA |
| NET4A_FW (1) | GGGGACAAGTTTGTACAAAAAAGCAGGCTTTATGGATTATGATCTGCTTCGTTC |
| NET4A_REV (2) | GGGGACAAGTTTGTACAAAAAAGCAGGCTTAAGAAGCAAGAATGGATGATG |
| NET4B_FW (3) | GGGGACAAGTTTGTACAAAAAAGCAGGCTTTATGGCTTCGTCTACGGCTCAG |
| NET4B_REV (4) | GGGGACAAGTTTGTACAAAAAAGCAGGCTTTCAAGTTGATAAGACCACTACTCTCTT |
